# CHST2-mediated sulfation of MECA79 antigens is critical for breast cancer cell migration and metastasis

**DOI:** 10.1038/s41419-023-05797-x

**Published:** 2023-04-24

**Authors:** Dan Zhang, Yihong Zhang, Xiuqun Zou, Mengying Li, Hui Zhang, Yaning Du, Jiamin Wang, Chicheng Peng, Chunyan Dong, Zhaoyuan Hou

**Affiliations:** 1 Hongqiao Institute of Medicine, Tongren Hospital/Faculty of Basic Medicine, Shanghai Jiaotong University School of Medicine, Shanghai, China; 2 Breast Cancer Center, Shanghai East Hospital, School of Medicine, Tongji University, Shanghai, China; 3 Shanghai Key Laboratory for Tumor Microenvironment and Inflammation, Department of Biochemistry & Molecular Cellular Biology, Shanghai Jiaotong University School of Medicine, Shanghai, China; 4 Shandong NARUI Biotechnology Co., LTD, Shandong, China

**Keywords:** Breast cancer, Cell migration

## Abstract

Snail is a denoted transcriptional repressor that plays key roles in epithelial-mesenchymal transition (EMT) and metastasis. Lately, a plethora of genes can be induced by stable expression of Snail in multiple cell lines. However, the biological roles of these upregulated genes are largely elusive. Here, we report identification of a gene encoding the key GlcNAc sulfation enzyme CHST2 is induced by Snail in multiple breast cancer cells. Biologically, CHST2 depletion results in inhibition of breast cancer cell migration and metastasis, while overexpression of CHST2 promotes cell migration and lung metastasis in nude mice. In addition, the expression level of MECA79 antigen is elevated and blocking the cell surface MECA79 antigen with specific antibodies can override cell migration mediated by CHST2 upregulation. Moreover, the sulfation inhibitor sodium chlorate effectively inhibits the cell migration induced by CHST2. Collectively, these data provide novel insights into the biology of Snail/CHST2/MECA79 axis in breast cancer progression and metastasis as well as potential therapeutic strategy for the diagnosis and treatment of breast cancer metastasis.

## Introduction

Breast cancer has become a primary cause of cancer mortality among women worldwide, and breast cancer metastasis accounts for the majority of cancer-related recurrences and deaths. During metastasis, cancer cells need to complete multistep cell-biological processes, which include matrix proteolysis and invasion into the tumor-associated stroma. Then, tumor cells enter into the lumina of lymphatic or blood vessels and survive in circulation. The final step involves in cancer cells crossing from the lumina vessel into the tissue parenchyma, forming micrometastases in distant organs, and finally proliferating within the microenvironment, leading to the formation of macroscopic metastases [1, 2]. Single-cell RNA sequencing has shown that breast cancer-derived liver and brain metastases exhibit a more complicated intratumoral heterogeneity and immunosuppressive ecosystem, which contribute to immune evasion and tumor malignancy [3]. Nevertheless, the molecular mechanisms underlying metastasis processes remain incompletely understood. As a consequence, further exploration of the metastatic biological processes in breast cancer will be critical for developing therapeutic interventions to combat breast cancer metastasis.

Epithelial-mesenchymal transition (EMT) is regulated by abundant factors, such as EMT transcription factors (Snail, Zeb, Twist), miRNA, circRNA and epigenetic regulators [4, 5]. Snail, in particular, is a well-studied transcription factor and a key regulator of EMT and cancer metastasis involved in multiple mechanisms [6]. Previous studies indicate that Snail promotes tumor progression by conferring tumor cells with stemness traits and promoting tumor cells’ chemoresistance, recurrences, metastasis and cancer-associated fibroblast activation [7–11]. Moreover, Snail can also prompt collective migration for tumor progression [12] and stimulate neurite outgrowth in prostate cancer progression [13].

Snail is denoted as a transcriptional repressor, largely conferred by the N-terminus SNAG (Snail/Gfi1) domain, which recruits multiple repressive complexes. However, several studies have shown that Snail can induce gene expression, in addition to transcription repression function. Snail has been shown to upregulate transcription of MMP9, ERCC1, MMP15, FN1, CLDN11 and PAPSS2 [12, 14–18]. In contrast to the thorough study on the mechanisms of Snail in transcription repression, little is known about the molecular mechanisms of Snail-mediated transcriptional activation and the biological role of these induced genes in cell migration and metastasis.

Carbohydrate sulfation of glycoconjugates has been shown to elicit diverse biological roles. The addition of a sulfate group on the glycan chains is catalyzed by various sulfotransferases. 6-O-Sulfation of N-acetylglucosamine (GlcNAc) residues, which occurs in N-linked and O-linked glycans, is catalyzed by a class of GlcNAc-6-O-sulfotransferase (GlcNAc6ST) [19]. G1cNAc6STs catalyze the transfer of sulfate group from 3′-phosphoadenosine 5′-phosphosulfate (PAPS) to the C-6 position of non-reducing GlcNAc. Thus far, five members of the GlcNAc6ST family have been cloned in humans [20], including CHST1 (chondroitin 6-sulfotransferase, keratan sulfate galactose-6-sulfotransferase, KSST), CHST2 (N-acetylglucosamine-6-O-sulfotransferase), CHST3 (chondroitin 6-sulfotransferase), CHST4 (HEC-GlcNAc6ST) and CHST5 (responsible for the synthesis of high-sulfated KS) [21, 22]. GlcNAc6ST-1 (CHST2) is a type II transmembrane protein consisting of a short N-terminal cytoplasmic tail, a hydrophobic single-pass transmembrane domain, an intervening stem region and a C-terminal catalytic domain that resides in the Golgi lumen [23]. CHST2 is widely expressed in high endothelial venules (HEVs) of various tissues [24], and is the first cloned GlcNAc6ST [25]. Previous studies have demonstrated that CHST2, along with CHST4, is critical for the biosynthesis of the 6-sulfo sialyl Lewis X epitope, which is recognized by the MECA79 antibody. In double-deficient CHST2/CHST4 (DKO) mice, the binding of the MECA79 antibody to l-selectin ligands expressed in the HEVs of peripheral lymph nodes was completely abrogated, leading to a 75% reduction in lymphocyte homing. These studies indicate that the essential role of CHST2 and CHST4 in lymphocyte homing and immune response [26–28]. However, the biology roles of CHST2 and MECA79 antigen in cancer progression remains poorly investigated and elusive.

In the present study we identify CHST2 as a Snail-induced gene and demonstrate that CHST2 plays critical role in Snail-mediated cell migration and metastasis by increasing cell surface MECA79 epitope synthesis.

## Results

### Snail induces CHST2 expression in breast epithelial cells

**Fig. 1:**
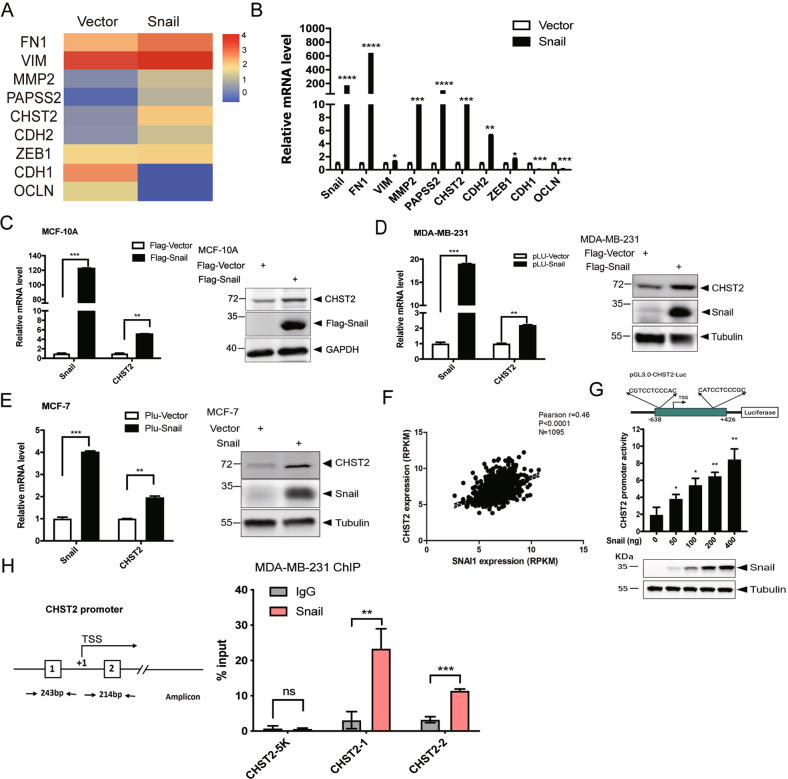
Snail induces the transcription and expression of CHST2 in breast cancer cells. **A** The heatmap showed the representatives of differentially expressed genes regulated by Snail in MCF-10A cells. **B** The known target genes regulated by Snail are validated by qRT-PCR. Data were shown as mean ± SD, ****P < 0.0001, ***P < 0.001, **P < 0.01, *P < 0.05, compared with vector group. Overexpression of Snail increased CHST2 mRNA levels in MCF-10A cells (**C**), MDA-MB-231 cells (**D**) and MCF-7 cells (**E**). Data were shown as mean ± SD, ***P < 0.001, **P < 0.01, compared with vector group (left panels). Western blots showed the protein expression levels of CHST2 in breast cancer cells with stably expressing Snail (right panels). **F** Scatter plots showed the correlation of Snail expression with CHST2 expression in 1095 breast tumor specimens from TCGA dataset. The *r* value was calculated via Pearson’s ranking correlation coefficient analysis. **G** Human CHST2-Luc promoter reporter activity was measured after co-transfection with Snail expression plasmids in 293T cells. Human CHST2 gene promoter (−638 to +426) was subcloned into a pGL3 basic luciferase vector to create a CHST2-Luc reporter construct (top panel). The results were expressed as normalized β-galactosidase activity. Error bars show standard deviations (middle panel) from three replicate experiments, **P < 0.01, *P < 0.05. **H** ChIP analysis of Snail binding to CHST2 promoter region in MDA-MB-231 cells. The ChIP assays were performed in MDA-MB-231 cells with specific monoclonal antibody against Snail and the relative enrichment of Snail on the CHST2 promoter region was determined by qPCR, the results are expressed as normalized input. Error bars show standard deviations. ***P < 0.001, **P < 0.01, *P < 0.05, ns means no significance. All the data are representative of at least three independent experiments and presented as the means ± SD.

To investigate the gene transcription profile mediated by Snail in breast cancer cells, we conducted RNA sequencing assays to analyze the gene expression profile in MCF-10A cells with stable Snail expression. Interestingly, the proportion of upregulated genes was similar to that of downregulated genes mediated by Snail [17]. The epithelial markers E-cadherin (encoded by CDH1) and Claudins (encoded by OCLN) were strongly repressed by Snail. Conversely, the mesenchymal markers N-cadherin (encoded by CDH2), Fibronectin (encoded by FN1) and Vimentin (encoded by VIM) were induced (Fig. 1A, B). Intriguingly, we observed the sulfation metabolism-related genes including PAPSS2 and CHST2 were obviously induced by Snail (Fig. 1A–C). These observations were further confirmed in MDA-MB-231 and MCF-7 cells (Fig. 1D, E). Consistent with our findings, analysis of The Cancer Genome Atlas (TCGA) database revealed a significant positive correlation between Snail and CHST2 expression levels in 1095 breast cancer specimens (Fig. 1F).

Next, to explore the molecular mechanisms underlying Snail’s regulation of CHST2 transcription, we inferred that CHST2 is a potential downstream target gene induced by Snail. As shown in Fig. 1G, two putative Snail-binding sites were identified in the CHST2 promoter region. Then we constructed luciferase reporter fusion plasmids containing the CHST2 promoter region encompassing the two binding sites (−638 to +426). To assess the effect of Snail on CHST2 promoter activity, the reporter plasmids were then transiently co-transfected with different doses of pcDNA3.1-Snail expression plasmids into 293T cells, and the promoter activities were measured using luciferase reporter assays. Indeed, Snail markedly induced CHST2 promoter reporter activities in a dose-dependent manner (Fig. 1G). To confirm that the two putative Snail-binding sites were necessary for Snail-mediated induction of CHST2 promoter activity, we mutated these two sites respectively and in-combination (GC-M1, GC-M2 and GC-M1,2). As shown in Supplementary Fig. 1A, B, the activation effect of Snail on the CHST2 promoter was abolished in the mutant constructs. These findings suggest that the two putative binding sites in the CHST2 promoter are essential for Snail-mediated activation effect on CHST2 transcription. Finally, to further determine whether Snail directly binds to the CHST2 promoter region, ChIP assays were performed in MDA-MB-231 cells and MCF-10A-Snail cells. The results showed that DNA fragment flanking the proximal promoter of CHST2 was pulled down by using antibody specifically against Snail (Fig. 1H, Supplementary Fig. 1C). Taken together, these data demonstrate that Snail transactivates CHST2 transcription through directly binding to the CHST2 promoter in breast epithelial cells.

### CHST2 promotes breast cancer cell migration and metastasis

**Fig. 2:**
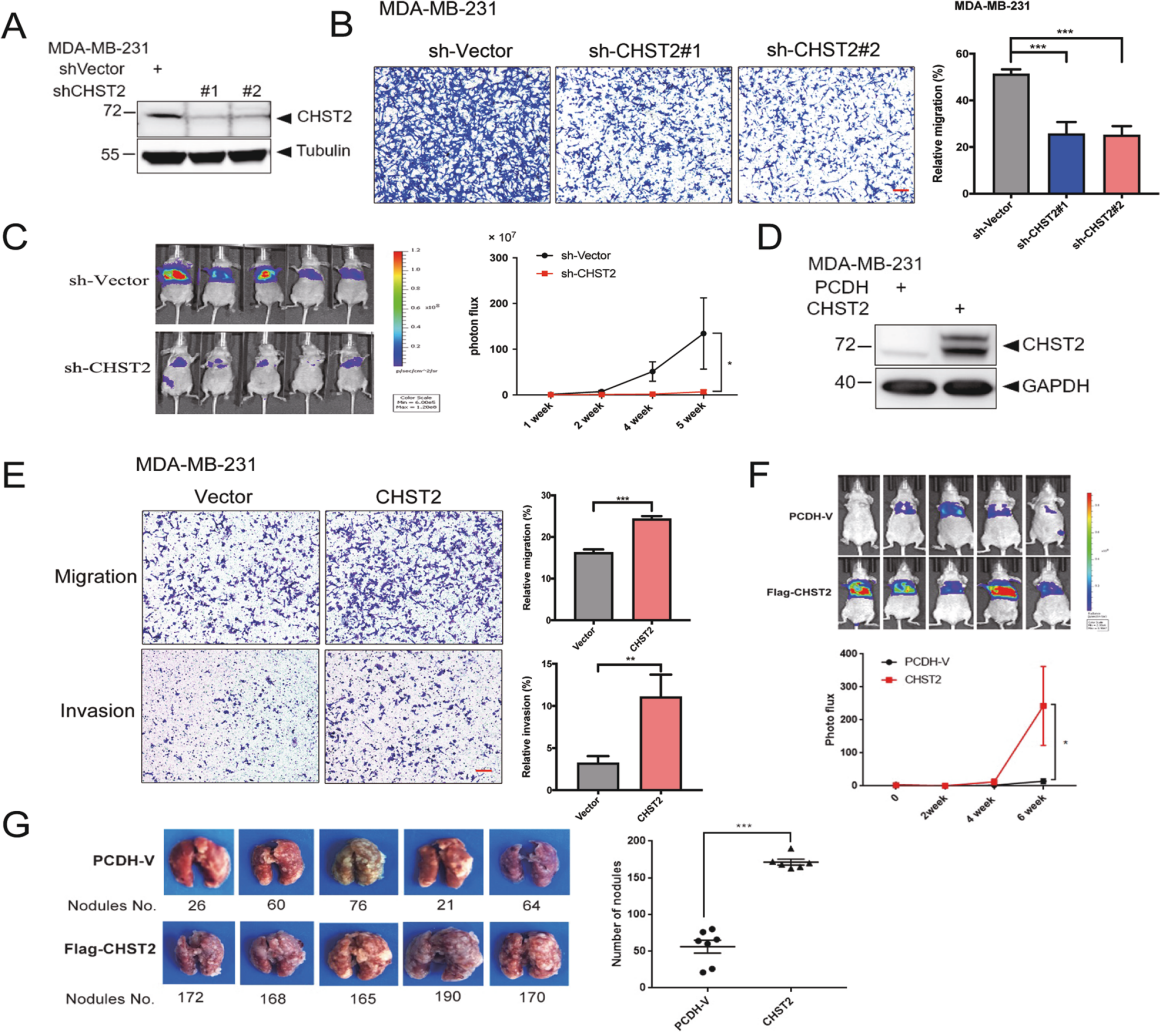
CHST2 is required for breast cancer cell migration and metastasis. Protein levels of CHST2 were quantified by western blots in MDA-MB-231 cells with CHST2 knockdown (**A**) or CHST2 overexpression (**D**). Transwell assays show the migration and invasion abilities of MDA-MB-231-shCHST2 cells (**B**) or MDA-MB-231-CHST2 cells (**E**). Left panel: Representative images of migrated cells, right panel: Statistical analysis of the average migrated cells per field. Data were shown as mean ± SD from three independent experiments. ***P < 0.001, **P < 0.01, compared with vector groups, 100×, scale bar, 100 μm. **C** Tail-vein injection of MDA-MB-231 cells with knockdown of CHST2 for lung metastasis analysis. Left panel: Intensities of lung metastasis in mice at the 5th week were analyzed by bioluminescent imaging (BLI). Right panel: Quantification of lung photon flux at the first, 2nd, 4th, and 5th week. *P* value was determined using two-way ANOVA, *P < 0.05, compared with vector group (*n* = 7 and *n* = 5, respectively). **F** Overexpression of CHST2 in MDA-MB-231 cells promoted lung metastasis in vivo. Left panel: Intensities of lung metastasis in mice at the 6th week were analyzed by BLI. Right panel: Quantification of lung photon flux at the 0, 2nd, 4th, and 6th week. *P* value was measured by two-way ANOVA, *P < 0.05, compared with vector group (*n* = 7 and *n* = 6, respectively). **G** Overexpression of CHST2 increased the number of metastatic pulmonary surface nodules. Left panel: Shown are representative images of metastatic nodules. Right panel: The average number of metastatic tumor nodules shows a significant difference between the vector and CHST2 overexpression group (*n* = 7 and *n* = 6, respectively). Data were shown as mean ± SD, ***P < 0.001, compared with vector group.

To explore the role of CHST2 in breast cancer progression, we first depleted CHST2 in MDA-MB-231 cells by stably expressing specific targeting shRNAs, subsequently, CHST2 protein levels were examined by Western blot assays (Fig. 2A). Transwell and invasion assays showed that the migration abilities of MDA-MB-231 cells with depleted CHST2 were markedly impaired (Fig. 2B). Conversely, stable expression CHST2 in MDA-MB-231 cells resulted in enhanced migration and invasion abilities (Fig. 2D, E). Furthermore, to examine whether CHST2 expression influences breast cancer cell growth, cell proliferation assays were conducted in breast cancer cells with CHST2 knockdown or overexpression. The results indicate that CHST2 expression has little effect on cell proliferation (data not shown).

To further explore the role of CHST2 in breast cancer metastasis in vivo, we performed tail-vein injection of MDA-MB-231 cells with vector control, CHST2 knockdown or overexpression into BALB/C nude mice to generate lung metastasis models. These cell lines were stably labeled with a luciferase reporter gene to facilitate lung metastasis quantification using the Bioluminescence Imaging System. The results showed lung metastasis of the CHST2 knockdown mice group grew slower compared with the vector control (Fig. 2C). To quantify lung metastasis, we measured the luciferase intensities at the 1rst, 2nd, 4th and 5th week after tail-vein injection (Fig. 2C, right panel). Notably, the mice group injected with MDA-MB-231-shCHST2 cells showed a markedly lower rate of lung metastasis compared with the vector group at the 4th and 5th week post tail-vein injection. In contrast, mice transplanted with CHST2 overexpression MDA-MB-231 cells exhibited higher lung metastasis compared to the vector control (Fig. 2F). Moreover, the number of metastatic pulmonary surface nodules significantly increased in MDA-MB-231-CHST2 mice compared to MDA-MB-231-Vector mice (Fig. 2G). Collectively, these findings demonstrated that CHST2 is essential for breast cancer cell migration and elevated CHST2 expression promotes breast cancer metastasis.

### CHST2 promotes cell migration in an enzymatic-dependent manner

**Fig. 3:**
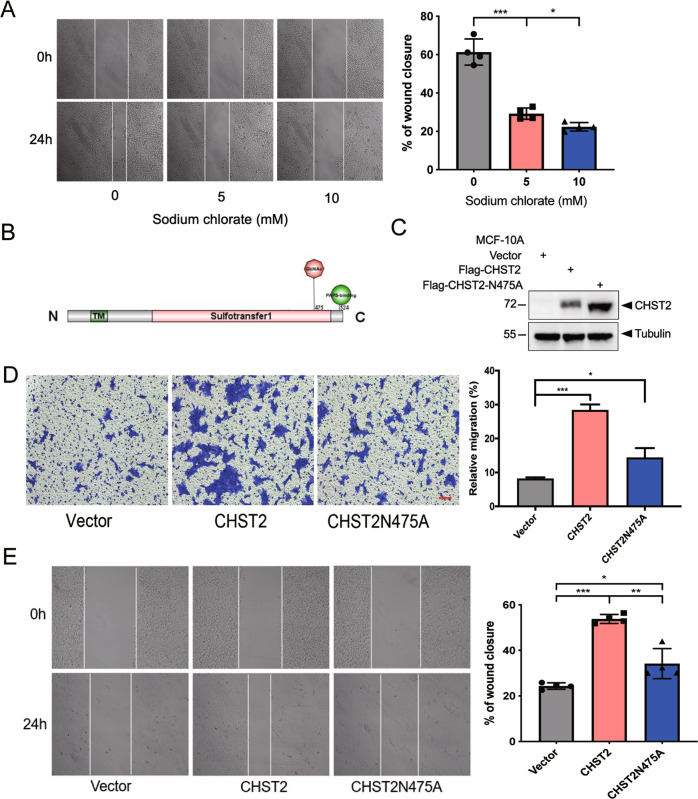
CHST2 promotes cell migration in an enzymatic-dependent manner. **A** The wound-healing assay showed migration capabilities of CHST2-overexpressed MCF-10A cells in the presence of 0, 5, 10 mM sodium chlorate for 48 h. Left panel: Representative images were shown. Right panel: The quantification of the percentage of wound closure. Data were shown as mean ± SD from four independent experiments, ***P < 0.001, *P < 0.05, Students *t* test. **B** The diagram of CHST2, the site of N475 which can be mono-glycosylated is critical for enzyme activity. **C** Western blots (WB) showed the protein level of MCF-10A-Vector, WT CHST2 and mutant CHST2-N475A cells in total cell lysates (TCL). **D** Transwell assays were performed in stable cell lines. Six fields chosen randomly were counted for statistical analysis. Data were shown as mean ± SD from three independent experiments. ***P < 0.001, *P < 0.05, Students *t* test. **E** Wound-healing assay showed the migration abilities of MCF-10A-Vector, CHST2 or mutant CHST2-N475A cells, left panel: Representative images were shown. Right panel: The quantification of the percentage of wound closure, Data were shown as mean ± SD. of four independent experiments, ***P < 0.001, **P < 0.01, *P < 0.05.

Next, we aimed to examine whether CHST2 promotes cell migration mediated by its enzymatic activity. The high-energy sulfate donor of PAPS is critical for CHST2 to catalyze the addition of sulfate groups to the GlcNAc. Therefore, we initially evaluated whether the PAPS deprivation affects the cell migration abilities mediated by CHST2. Wound-healing assays showed cell migration mediated by CHST2 overexpression MCF-10A cells markedly decreased in the presence of sodium chlorate (Fig. 3A), a selective inhibitor in the synthesis of high-energy donor of sulfate-PAPS (3′-phosphoadénosine 5′-phosphosulfate) [28, 29]. Furthermore, we overexpressed CHST2 in MCF-7 cells and administrated with sodium chlorate, and transwell assays showed concurrent effects in inhibition of cell migration with sodium chlorate treatment (Supplementary Fig. 2A, B). In addition, at the concentration of 5 and 10 mM sodium chlorate without apparently affecting cell viabilities and CHST2 protein expression levels (Supplementary Fig. 2C, D, Supplementary Fig. 3B) [17].

The amino acid residue (Asn 475) is a key N-linked glycosylation site of CHST2, which can be mono-glycosylated by the addition of glycosyl GlcNAc. Figure 3B shows CHST2 diagram. Previous studies have noted that CHST2 is unable to sulfate its substrate sialyl Lewis X tetrasaccharide upon mutation of the Asn 475 residue, indicating that the N475 residue is critical for CHST2 catalytic activities [30, 31]. We therefore investigated whether CHST2 enzyme activity affects its function in facilitating breast cancer migration. We infected MCF-10A cells with vector, wild-type (WT) or catalytically inactive mutant (N475A) CHST2, and their expression levels were examined by Western blotting. The results showed that the mutant CHST2-N475A band was lower than that of the wild-type, likely due to N-glycosylation of N475 disruption (Fig. 3C). Cell migration assays revealed that overexpression of CHST2-N475A led to a slight increase in cell migration rate compared with the vector control but was markedly lower than the cells bearing wild-type CHST2 (Fig. 3C, D). Additionally, wound-healing assays further confirmed that CHST2 overexpression obviously increased cell migration capacity, while MCF-10A with CHST2-N475A overexpression had only a slight increase in cell migration (Fig. 3E). Together, these data demonstrate that sulfotransferase activity is required for CHST2 to induce cell migration.

### MECA79 antigen is an obligate downstream target to mediate CHST2-induced breast cancer metastasis

**Fig. 4:**
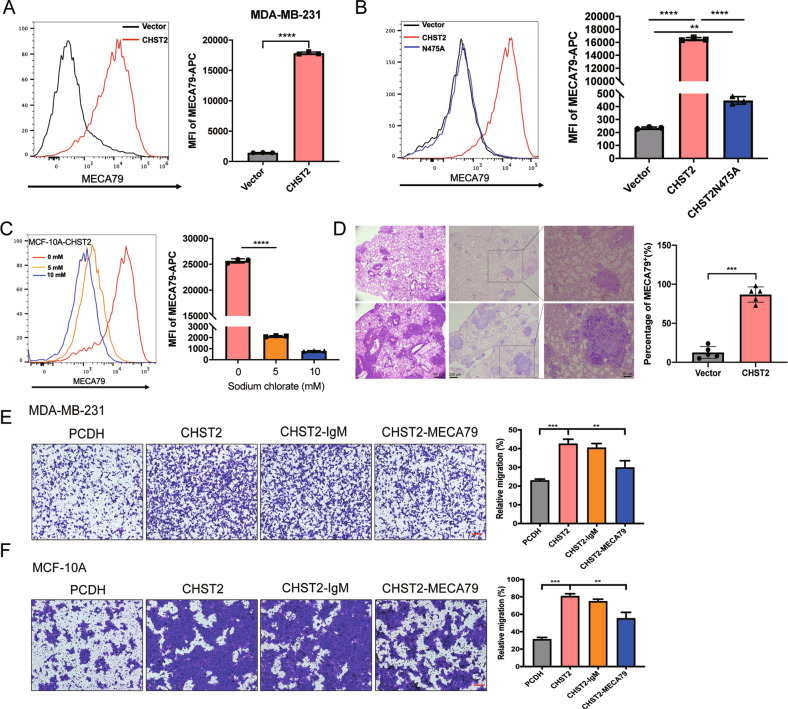
CHST2 is involved in MECA79 antigen synthesis to mediate breast cancer metastasis. **A** FACS analysis of MDA-MB-231-Vector (black) and MDA-MB-231-CHST2 (red) cells stained by MECA79 mAb, left panel: Representative histograms were shown. Right panel: MFI of MECA79 antigen synthesis by breast cancer cells was shown compared with vector group, MFI, mean fluorescence intensity, the MFI of MECA79 less the MFI of isotype control equals the Delta MFI. Data were shown as mean ± SD, ****P < 0.0001, Student *t* test. **B** Flow cytometry analysis of the MECA79 surface expression in MCF-10A-Vector (black), CHST2 (red) or mutant CHST2-N475A (blue) cells, left panel: Representative histograms were shown. Right panel: Relative MFI of MECA79 antigen synthesis by MCF-10A cells was shown compared with the vector group. Data were shown as mean ± SD from three independent experiments. ****P < 0.0001, **P < 0.01, Student *t* test. **C** Flow cytometry analysis of the MECA79 surface expression in MCF-10A-CHST2 cells with treatment of 0, 5, 10 mM sodium chlorate for 48 h, left panel: Representative histograms were shown. Right panel: MFI of MECA79 antigen synthesis by MCF-10A-CHST2 cells was shown. Data were shown as mean ± SD from three independent experiments. ****P < 0.0001, Student *t* test. **D** H&E staining and Immunohistochemical staining of MECA79 antigen of the lung sections isolated from lung metastatic mice models. **E**, **F** Transwell assays in MDA-MB-231-Vector or MCF-10A-Vector and MDA-MB-231-CHST2 or MCF-10A -CHST2 cells with MECA79 or isotype antibody treated (left panel), Six fields chosen randomly were counted for statistical analysis (right panel) and data were shown as mean ± SD, ***P < 0.001, **P < 0.01. Student *t* test.

To investigate the mechanisms underlying CHST2-mediated promotion of breast cancer metastasis. Here, we examined the CHST2 substrate in breast cancer cells. Flow cytometric analysis of MDA-MB-231-CHST2 cells showed that CHST2 overexpression significantly increased cell surface MECA79 expression compared to the vector control (Fig. 4A). Similarly, the results in MCF-10A-CHST2 cells also displayed higher expression of MECA79 antigen compared to the vector control (Fig. 4C), while in CHST2-N475A mutant cells the MECA79 antigen expression was not apparently increased (Fig. 4B). These observations imply that CHST2 is involved in the synthesis of MECA79 antigen in breast cancer cells. Additionally, flow cytometric analysis of CHST2-overexpressed MCF-10A cells demonstrated that MECA79 antigen synthesis was diminished with sodium chlorate treatment (Fig. 4C). Notably, the synthesis of MECA79 antigen was markedly increased in CHST2 overexpression MDA-MB-231 metastatic lung sections (Fig. 4D). These findings demonstrated that CHST2 is involved in MECA79 antigen synthesis in breast cancer cells and that MECA79 antigen is a potential downstream functional mediator of CHST2’s effects on cancer cell migration.

Furthermore, in order to determine whether cell surface MECA79 antigen synthesis by CHST2 is responsible for mediating breast cancer cell migration and to evaluate whether targeting MECA79 antigen could be an effective strategy in inhibition of cancer cell migration. We employed an MECA79 antibody as an antagonist, and consistently, CHST2-mediated cell migration decreased markedly by blocking mAb MECA79 to cell surface MECA79 antigen in MDA-MB-231 and MCF-10A cells (Fig. 4E, F). Collectively, these data suggest that CHST2-mediated breast cancer cell migration and metastasis are dependent on cell surface MECA79 antigen synthesis.

### CHST2 is an essential target gene for Snail-induced cell migration

**Fig. 5:**
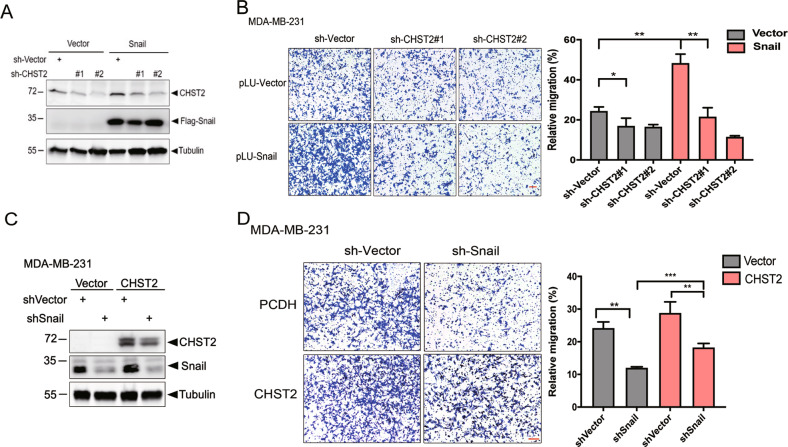
CHST2 is an essential target gene for Snail-induced cell migration. Western blots showed the protein levels of Snail, CHST2. Knocking-down of CHST2 in Snail overexpression MDA-MB-231 cells (**A**) and Snail knockdown with CHST2 rescued cells (**C**). Transwell assays in MDA-MB-231-pLU-Snail cells (**B**) and MDA-MB-231-shSnail cells (**D**) with CHST2 knockdown and CHST2 rescue respectively. Three independent experiments were performed, six fields chosen randomly were counted for statistical analysis (right panels) and data were shown as mean ± SD, ***P < 0.001, **P < 0.01, *P < 0.05. Students’ *t* test.

To determine whether CHST2 is a critical target for Snail-induced breast cancer metastasis. We depleted CHST2 in MDA-MB-231-Vector and MDA-MB-231-Snail cells (Fig. 5A), and cell migration assays were performed. Consistently, Snail overexpression accelerated cell migration, while CHST2 depletion overrode Snail’s effects on mediating cell migration in MDA-MB-231 cells (Fig. 5B). Furthermore, deprivation of PAPS with sodium chlorate treatment reduced Snail-mediated cell migration (Supplementary Fig. 3A). Conversely, cell migration can be partially rescued in MDA-MB-231-shSnail cells with CHST2 overexpression (Fig. 5C, D). Considered together, these data demonstrate that CHST2 is an important downstream target of Snail and that elevated CHST2 expression is critical for Snail-induced cell migration.

### Snail-CHST2 axis mediated migration of breast cancer cells by enhanced MECA79 antigen synthesis

**Fig. 6:**
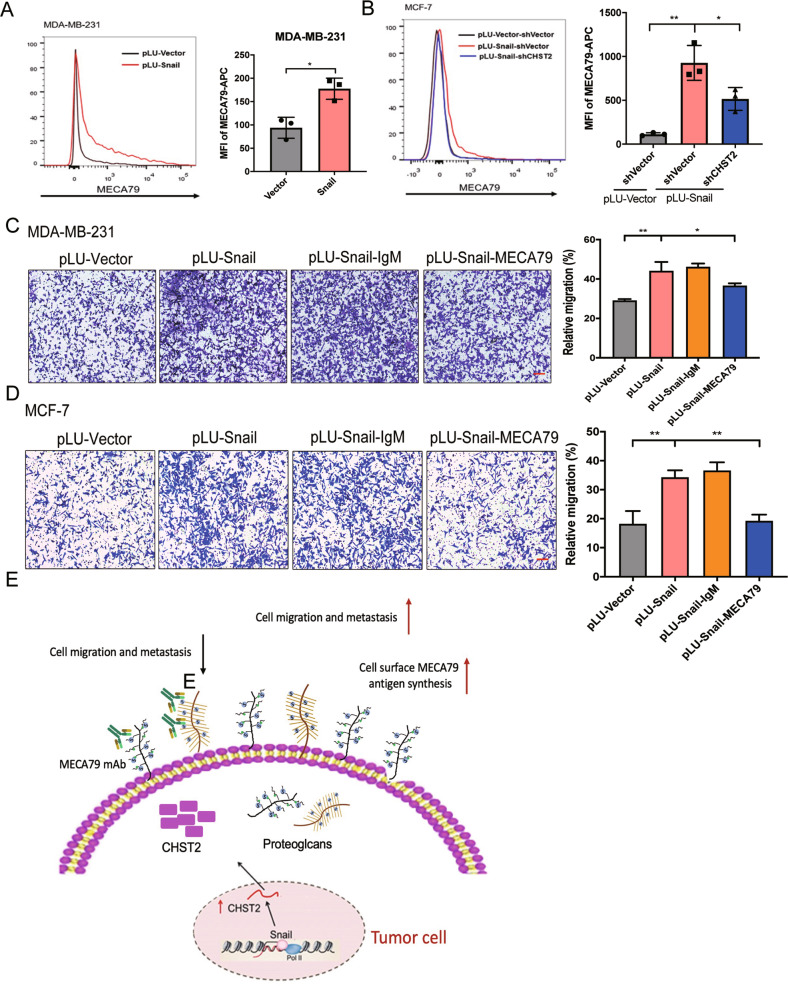
Snail-CHST2 axis mediated breast cancer cell migration by enhanced MECA79 antigen synthesis. **A** FACS analysis of MDA-MB-231-pLU-Vector (black) and MDA-MB-231-pLU-Snail (red) cells stained by MECA79 mAb, left panel: Representative histograms were shown. Right panel: MFI of MECA79 antigen synthesis by breast cancer cells was shown compared with the vector group. Data were shown as mean ± SD from three independent experiments. *P < 0.05, Student *t* test. **B** MECA79 surface expression in MDA-MB-7-pLU-Vector (black) cells and MCF-7-pLU-Snail cells with CHST2 knockdown was assessed by flow cytometry, left panel: Representative histograms were shown. Right panel: MFI of MECA79 antigen synthesis by cancer cells was shown compared with Snail group. Data were shown as mean ± SD, three independent experiments were performed. **P < 0.01, *P < 0.05, Student *t* test. **C**, **D** Transwell assays in MDA-MB-231-Vector or MCF-7-Vector and MDA-MB-231-Snail cells or MCF-7-Snail cells with MECA79 or isotype antibody treated (left panels), six fields chosen randomly were counted for statistical analysis (right panels) and data were shown as mean ± SD of three independent experiments, **P < 0.01, *P < 0.05. Student *t* test. **E** Schematic model for the role of Snail-CHST2-MECA79 sulfation axis in breast cancer cell migration and metastasis. Snail activated transcription of CHST2 via binding to promoter sequences, and simultaneously induced CHST2 expression which increased sulfation level in MECA79 antigen synthesis to enhance migration and metastasis of breast cancer cells. Furthermore, blocking cell surface MECA79 antigen inhibited cancer cell migration.

Our results have demonstrated that CHST2 facilitates cell migration through the synthesis of MECA79 antigen and acts as an essential target of Snail, which is critical for Snail-triggered cell migration. To determine whether Snail can increase MECA79 antigen level in breast cancer cells, flow cytometric analysis of MDA-MB-231-Snail cells and MCF-7-Snail cells were carried out. Indeed, Snail overexpression enhanced MECA79 synthesis (Fig. 6A, B), which was dampened by concurrent CHST2 depletion (Fig. 6B). These results suggested that MECA79 antigen synthesis is elevated with overexpression of Snail, which is dependent on CHST2 expression.

To determine whether the Snail-CHST2 axis promotes breast cancer migration through mediating cell surface MECA79 antigen synthesis. MECA79 antibody was added in stably Snail overexpression breast cancer cells, and consistently, we observed a significant reduction in Snail-mediated cell migration in both MDA-MB-231 and MCF-7 cells (Fig. 6C, D). Taken together, these data suggest that the Snail-CHST2 axis mediated breast cancer migration by increasing MECA79 antigen synthesis. Furthermore, the MECA79 antigen may serve as a tumor surface antigen and could be a potential target for inhibiting tumor metastasis.

## Discussion

Snail as a classic transcription repression factor can recruit a variety of repressive complexes to the N-terminal SNAG domain, these complexes act as Snail corepressors to exert transcription repression function through epigenetic regulation. Commonly, Snail directly bind with various epigenetic regulatory proteins to regulate genome epigenetic modifications [32–35]. The molecular mechanisms of Snail-mediated target gene repression have been thoroughly elucidated. However, mechanisms underlying Snail-mediated transcriptional activation remain poorly investigated. Preliminary studies have shown that Snail can induce MMP9, ERCC1, MMP15, FN1, CLDN11 and PAPSS2 transcription and expression through direct or indirect regulations [12, 14–18]. In the present study, we illustrate that Snail can activate considerable genes transcription in breast epithelial cells (Fig. 1A). Especially, Snail induces CHST2 transcription and expression through directly binding to its promoter sequences. And the activation effect of Snail on CHST2 promoter is abolished in both of the single site mutant constructs, we postulate that binding to one site by Snail cannot drive the transcription of CHST2 gene, and requires the presence of two or more binding sites. The underlying mechanism remain an interesting question which will be addressed further.

High expression of CHST2 results in increased sulfation modifications of glycan determinants in breast cancer cells. The sulfation substrate 6-sulfo sialyl LewisX can be recognized by MECA79 monoclonal antibody which is also called MECA79 antigen. Usually, 6-sulfo sialyl LewisX is decorated on the l-selectin ligands expressed in high endothelial venules (HEVs) in lymph nodes or the HEV-like vessels at sites of chronic inflammation to mediate lymphocyte homing or leukocyte rolling [28]. Clinical investigations demonstrated the ectopic expression of the MECA79 antigen in tumor tissues may facilitate cancer cell metastasis [36]. However, the role of CHST2-mediated sulfation process in breast cancer has not yet been reported. In our study, we identified a novel sulfation pathway regulated by Snail-CHST2-MECA79 axis may contribute to breast cancer metastasis.

Previous work showed CHST2-deficient mice exhibit reduced synthesis of 6-sulfo sialyl Lewis X in HEVs, leading to impaired lymphocyte homing [24]. Besides, CHST2 is involved in synthesizing keratan sulfate, which facilitates glial scar formation after brain injury or 5D4 KS epitope expression in early postnatal brains [37, 38]. Furthermore, Kenji Uchimura et al. have reported that CHST2 exhibits higher expression in human tumor cells, such as leukemia cells and lymphoma cells [39]. In this study, we report that the expression of CHST2 contributes to breast cancer metastasis. We discovered breast cancer cell migration and metastasis reduced after depleted CHST2, while cell proliferation is not affected. Conversely, overexpression of CHST2 increased breast cancer cell migration and metastasis in vitro and in vivo. Moreover, cell migration assays indicated that CHST2 is required in Snail-mediated cell metastasis. It is known that in many cellular types overexpression of Snail induces EMT and cell migration, but how much role of EMT contributes to cell migration is unknown. Besides, other mechanisms exist for Snail-mediated cell migration, including formation of lamellipodia upon activation Rac1 and the promotion of neurite outgrowth in prostate cancer[13, 40]. In this study, we discovered CHST2 acts as a novel downstream target to promote Snail-mediated cell migration by increasing cell surface MECA79 antigen synthesis. In addition, sodium chlorate treatment or catalytically inactive mutant can efficiently abolish CHST2 or Snail-mediated cancer cell migration. Those results suggest that CHST2 acts as a critical role in breast cancer metastasis and its function in mediating sialyl LewisX sulfation is responsible for breast cancer metastasis.

The biosynthesis of 6-sulfo sialyl LewisX is a multienzyme catalytic process of glycosylation and sulfation, the mechanisms of glycosylation and post-translational modifications of carbohydrate structures are extremely intricate. Sulfation at the non-reducing C-6 of GlcNAc is essential for L-selectin binding and MECA79 epitope synthesis [41]. Here, we focused on the ubiquitously expressed enzyme CHST2 which catalyzed the transfer of sulfate groups to GlcNAc structures. Our experiments clearly show that CHST2 is responsible for MECA79 antigen synthesis in breast cancer cells. Furthermore, Snail-CHST2 axis mediated sulfation of sialyl LewisX is probably the downstream target to facilitate breast cancer metastasis. The results account for clinical studies which showed ectopic expression of MECA79 antigen on tumor specimens are linked with poor prognosis and metastasis [36, 42, 43]. The carbohydrate antigen sialyl LewisX is a terminal carbohydrate structure which is associated with tumor malignancy. Sialyl LewisX can serve as a tumor marker and alter cancer cell phenotypes by increasing cancer cell motility and proliferation through E-selectin–mediated cancer cell adhesion to vascular endothelial cells or E-selectin-independent pattern [44, 45]. 6-sulfo sialyl LewisX is the sulfation form of sialyl LewisX, in which the GlcNAc is sulfated by specific sulfotransferases. The sulfation modification alters the binding and expression properties of sLeX, making it a ligand for L-selectin, a cell surface receptor expressed on leukocytes [24, 46]. Overall, both sialyl LewisX and 6-sulfo sialyl LewisX are carbohydrate antigens that are associated with tumor malignancy and play important roles in cancer progression and metastasis [47]. However, 6-sulfo sialyl is less well understood in terms of its role in cancer biology. In our study, we tried to elucidate the biological role of 6-sulfation sialyl LewisX in breast cancer and discovered cell surface 6-sulfo sialyl LewisX synthesis mediated by CHST2 is associated with breast cancer metastasis and may be a potential novel breast cancer metastatic marker.

Current findings demonstrate Snail induces CHST2 transcription, which results in cell surface 6-sulfo sialyl LewisX synthesis increase and facilitates migration and metastasis of breast cancer cells. Nevertheless, further studies must be done in the future to elucidate the detailed mechanisms of ectopic MECA79 antigen expression in breast cancer. Previous studies have reported that glycoproteins carrying 6-sulfo sLeX include GlyCAM-1, CD34, MAdCAM-1, PODXL and EMCN, a class of proteins that have mucin-like domains, which act as scaffoldings for O-linked oligosaccharides [48, 49]. Therefore, further investigations will be carried out to identify the glycoproteins and to elaborate on the mechanisms of MECA79 synthesis in breast cancer.

Indeed, studies have demonstrated that 6-sulfo sLeX synthesis in L-selectin ligands requires two 6-sulfotransferases, N-acetylglucosamine-6-O-sulfotransferase-2 (GlcNAc6ST-2, CHST4) which specifically restricted expressed in HEV and ubiquitously expressed CHST2 [22]. In the present study, we have revealed that the expression of CHST2 is induced by Snail in breast cancer cells, while CHST4 expression is not affected by Snail. It has been reported that CHST2 and CHST4 mediated sulfation of sLeX are tissue- and substrate-specific. Specifically, Kenji Uchimura et al. reported CHST2 is the major sulfotransferase enzyme in Peyer’s patches, while in peripheral and mesenteric lymph nodes, CHST4 plays a key role in the synthesis of 6-sulfo sLeX. Those results suggest that CHST2 and CHST4 play complementary roles in 6-sulfo sLeX synthesis in lymph nodes [38]. Here, we reported that Snail-induced expression of CHST2 in breast cancer cells is responsible for 6-sulfo sLeX synthesis, which mediates breast cancer metastasis.

In conclusion, our observations shed light on the molecular mechanisms underlying Snail transactivating CHST2 transcription and expression and elaborate the role of Snail-CHST2 axis in mediating sulfation of sialyl LewisX on the cell surface during breast cancer metastasis. These results provide new insights into Snail function in cancer metastasis. Furthermore, this novel sulfation pathway regulated by Snail may be a promising therapeutic strategy by specifically targeting the MECA79 antigen. In addition, the ectopic expression of the MECA79 antigen will contribute to the diagnosis and treatment of breast cancer.

## Materials and methods

### Cells and cell culture

The human breast cancer cell lines MDA-MB-231, MCF-7 and non-tumorigenic mammary cells MCF-10A were originally obtained from the American Type Culture Collection (ATCC). Human embryonic kidney cell line 293T, MDA-MB-231 and MCF-7 cells were cultured in Dulbecco’s modified Eagle’s medium (DMEM) supplemented with 10% fetal bovine serum (FBS), 2 mM l-glutamine and penicillin (50 U/ml)/streptomycin (50 μg/ml). MCF-10A cells were maintained in DMEM/F12 supplemented with 5% horse serum, insulin (10 μg/ml), hydrocortisone (0.5 μg/ml), EGF (20 ng/ml), cholera toxin (100 ng/ml) and penicillin/streptomycin. The cell lines were authenticated by DNA fingerprinting in the Shanghai Jiao Tong University Analysis Core and were cultured in a 37 °C water-saturated 5% CO_2_ humidified chamber.

### Plasmids

The human CHST2 cDNA was subcloned into pCDH-CMV-MCS-EF1-Puro-vector by PCR technique with EcoR I and Not I restriction enzyme sites. The pCDH-Flag-CHST2-N475A variant were cloned from pCDH-Flag-CHST2 by point mutation of the amino acid residue 475 N into A. The CHST2 gene promoter spanning from −638 to +426 was generated from genomic DNA of the human embryonic kidney cell line 293T by PCR and subcloned into pGL3.0-Luc basic vector, generating the pGL3-CHST2-Luc reporter constructs. The pLU-Flag-Snail plasmid has been previously described [34]. The short hairpin RNA sequences of pLKO.1- shCHST2 were obtained from GE-Life science and are listed in Table 1.

**Table 1:** Primer list.

	Primers	Sequences
Sense sequences in shRNA	CHST2 shRNA-1	ATACAACTGGAAGACAGAGAG
	CHST2 shRNA-2	TTTGATCTGCTGGAAGGTGAG
Primers for ChIP	CHST2-1 Forward	GCCACAGACCCACTAGAGAG
	CHST2-1 Reverse	GAGAGTTGGATTCTGCGTGC
	CHST2-2 Forward	CACCTGGGTTTCCTTGCCTA
	CHST2-2 Reverse	GAGAGGTGGGAAAATTCGCC
	CHST2-5k Forward	CAATCAAGCCCAAGGAAAGA
	CHST2-5k Reverse	ACAGGCTGGCTTACTGCCTA
Primers for qRT-PCR	Snail-84-Forward	TTTACCTTCCAGCAGCCCTA
	Snail-291-Reverse	CCCACTGTCCTCATCTGACA
	CHST2-1322- Forward	TTTTGTGGGACTGTTGGTGA
	CHST2-1485- Reverse	CACCTGTTTGATCTGCTGGA

### RNA extraction and RT-PCR analysis

Total RNA was extracted following standard protocols with TRIzol reagent (Ambion, Carlsbad, CA, USA). Complementary DNAs were synthesized with 3 μg of total RNA using iScript cDNA Synthesis Kit (Fermentas, San Jose, CA, USA). The detailed procedures of RNA extraction and qRT-PCR were previously described [50]. The qRT-PCR assays were performed with SYBR green reagent (ABI 7500 Fast).

### Western blots and antibodies

Protein samples were prepared from various cells at 60~80% confluency. The cells were washed in ice-cold PBS, and then lysed in ice-cold lysis buffer containing protease inhibitor phenylmethylsulfonyl fluoride (PMSF) and cocktail, 25 mM Tris-HCl (pH 7.4), 150 mM NaCl, 0.5% NP40, 1 mM EDTA. Cell lysates were then clarified by centrifugation, protein concentrations were determined using the bicinchoninic acid (BCA) Protein Assay Kit (Thermo Scientific, USA). The antibodies were used in western blots as follows: CHST2 antibody (26027-1-AP, Proteintech, Chicago, IL, USA), Snail antibody (3879S, Cell Signaling Technology, Massachusetts, USA), anti-Flag (F3165, F7425, Sigma, St. Louis, MO, USA), Alpha Tubulin antibody (66031-1-Ig, Proteintech, Chicago, IL, USA), GAPDH antibody (60004-1-Ig, Proteintech, Chicago, IL, USA), Normal rabbit IgG (sc-2027, Santa Cruz).

### Transfection, luciferase reporter assays and ChIP

For transfection, 293T cells were seeded at a density of 5 × 10^4^ cells per well in 24-well plates. The β-galactosidase plasmids (10 ng) and pGL3-CHST2-Luc reporter (100 ng), along with Snail-encoding plasmids, were transiently transfected into the cells with Lipofectamine 3000 reagent (Invitrogen, Carlsbad, CA). The following procedures were performed as described [34]. ChIP assay was performed according to published protocols [34] in MDA-MB-231 cells and MCF-10A stably expressing Snail cells with slight modifications. To prepare cells for ChIP, MDA-MB-231 cells and MCF-10A-Snail cells were grown in 100-mm plates to 70–80% confluence and fixed by the addition of 287 μl of 37% formaldehyde directly into 10 ml of growth medium to a final concentration of 1% for 15 min at room temperature. The cross-linking reaction was stopped by the addition of 625 μl of 2 M glycine in phosphate-buffered saline buffer at room temperature for 5 min. 1 × 10^7^ cells were harvested, the chromatin was sonicated into fragments ranging from 500 to 800 bps in size, the sonication fragments were resolved on 2% agarose gels and visualized with ethidium bromide. The immunoprecipitated DNAs were amplified by real-time PCR with primer sets listed in Table 1.

### Cell migration and invasion assays

Migration of breast cancer cells was determined using 24-well Boyden chambers (Corning) with 8 μm-inserts. The detailed procedures of migration assay were previously described [17, 51]. Exponentially growing MDA-MB-231, MCF-10A or MCF-7 cells were digested by trypsin and collected by centrifugation. The cells were resuspended in serum-free DMEM or DMEM/F12 blank medium and counted. 2.5 × 10^4^ of MDA-MB-231 or 5 × 10^4^ of MCF-10A or 4 × 10^4^ of MCF-7 cells in 100 μl serum-free DMEM or DMEM/F12 blank medium were seeded on 8 μm-inserts, then 600 μl DMEM or DMEM/F12 complete growth medium were added to the bottom chamber as attractants. After incubation for 20–24 h, the cells were fixed with 4% paraformaldehyde and stained with crystal violet, non-migrated cells on the top of the chamber were removed gently with cotton swabs, then migrated cells were counted as per field of view under phase-contrast microscopy. For invasion assay, the matrigel matrix (Corning, #354234) was diluted to 250 μg/ml on ice, and 100 μl diluted matrigel was added into 8 μm-inserts. Then the transwell filter was placed in the 37 °C incubator for 2 h until the matrigel solidified. The following procedures are similar to migration assays. The number of migrated cells was calculated by the relative cell migration area through Image-J software.

### Wound-healing assays

MCF-10A cells were stably expressed with Flag-CHST2 and Flag-CHST2-N475A individually, then, subconfluent growing cells were digested by trypsin and counted using hemocytometer, 5 × 10^5^ cells were seeded onto a 6-well tissue culture plate. After incubating the seeded cells for 6 h at incubator with complete DMEM/F12 medium, the confluent monolayer cells were scratched in a straight line to create a “scratch” with a 10 μl pipette tip, removing the cell debris and smooth the edge of the scratch by washing the cells twice with 2 ml of the PBS, and then added 2 ml DMEM/F12 medium supplemented with 1% horse serum, insulin (2 μg/ml), hydrocortisone (0.1 μg/ml), EGF (4 ng/ml) and cholera toxin (20 ng/ml). And afterwards, creating markings on the outer bottom of the dish with an ultrafine tip marker. Then 0 h images were captured using a phase-contrast microscope. Place the plates in incubator at 37 °C under 5% CO_2_ for 24 h, the second images were captured of the same wounds at 24 h. The scratch areas were measured and presented as the percentage of scratch areas at 0 h (mean ± SD). The percent of migration was calculated as the migration value of the scratch areas at 24 h divided by the scratch areas at 0 h. Each sample was performed at least three times [52].

### Cell proliferation and cell viability

Cell proliferation rate and cell viability were measured by cell counting kit-8 (C0038, Beyotime). For cell proliferation analysis, seed cells in a 96-well plate at a density of 1 × 10^3^ cells per well in 100 μl of culture medium and incubate the plate in the incubator for ~12 h, then add 10 μl of CCK8 solution to each well and incubate another 1 hour, measure the absorbance at 450 nm every other day to determine the cell proliferation rate. For cell viability assay, 5 × 10^3^ cells were seeded onto a 96-well plate, then incubate the plate for 4~6 h in a humidified incubator at 37 °C, 5% CO_2_, and after cells were treated with 0, 5 and 10 mM sodium chlorate for 24 h, add 10 μl of CCK8 solution to each well with another 1-hour incubation in the incubator, the absorbance of 450 nm was tested to determine cell viabilities.

### Mouse experiments

Female BALB/c nude mice were purchased from SLAC laboratory Co. Ltd (Shanghai, China) and divided into two groups randomly. To establish in vivo lung metastatic mouse models of MDA-MB-231 cells, Exponentially growing MDA-MB-231 cells (labeled with luciferase reporter) were digested and resuspended in PBS buffer, then 1 × 10^6^ of MDA-MB-231-sh-CHST2/sh-Vector or 5 × 10^5^ of MDA-MB-231-Flag-CHST2/Vector cells in 100 μl of PBS suspensions were injected into the tail vein of 6-week-old mice. Lung micro-metastatic foci were analyzed by using the Xenogen IVIS Imaging System (PerkinElmer). Animal studies were conducted following the Institutional Animal Care and Use Committee of Shanghai in accordance with the National Research Council Guide for Care and Use of Laboratory Animals (SCXK, Shanghai 2007-0005). All mice were fed under specific pathogen-free (SPF) conditions. To ameliorate any suffering, mice were euthanized by CO_2_ inhalation.

### Hematoxylin-eosin staining and immunohistochemistry assays

After lung metastatic mice models were analyzed at the endpoint, the mice were euthanized and lungs with metastatic foci were dissected and fixed with 5 ml of 4% paraformaldehyde. Tissues were then embedded in paraffin blocks, and sections were cut (4 μm) for H&E staining and immunohistochemistry. For immunohistochemical staining, lung sections were baked at 65 °C overnight, then de-paraffinized by three 10-min extractions in xylene, followed by a series of steps de-paraffinized using 100%, 100%, 90%, 90% and 75% ethanol for 5 min each. Then tissue slides are soaked in gently running tap water for 30 min. Afterwards, sections were transferred to boiling sodium citrate buffer (pH 6.0) for antigen retrieval for 30 min and then cooled to room temperature. Then, sections were pre-treated with 3% hydrogen peroxide for 15 min before blocking. Blocking was performed with 5% bovine serum albumin in PBS for 30 min at room temperature followed by primary antibody incubation overnight at 4 °C. Immunoreaction was detected using SABC-AP assay (Boster, SA1050) according to the manufacturer’s instructions. The sections were visualized under a microscope. The following primary antibodies were used: rat monoclonal MECA‐79 antibody (sc‐19602, 1:50; Santa Cruz Biotechnology, Santa Cruz, CA).

### Flow‑cytometric analysis

MDA-MB-231, MCF-7 and MCF-10A cells were harvested at a subconfluent stage, then the cells were collected by centrifugation for 5 min at 800 × *g*, at 4 °C, washed twice and resuspended in PBS containing 1% FBS at 1 × 10^6^ cells/ml density. Subsequently, the cell suspensions were incubated with each mAb (MECA79 (rat mAb, Santa Cruz Biotechnology) or normal rat IgM (sc-3885, Santa Cruz Biotechnology) for 30 min on ice, followed by staining with APC-conjugated goat anti-rat IgM (A10540, Invitrogen, Carlsbad, CA, USA) or FITC-conjugated sheep anti-rat (PA1-28638, Invitrogen, Carlsbad, CA, USA) IgG for 30 min on ice, and then analyzed on a FACScan (CytoFLEX LX, Beckman Coulter, USA). Experiments were performed in triplicate.

### Statistical analysis

Statistical analysis was performed using SPSS 19.0 (IBM Corp., Armonk, NY, USA) and Graph Pad Prism 7.0 (Graph Pad Software, Inc., La Jolla, CA, USA) software. The independent student’s t tests were used for comparison between two groups. Data are presented as the mean ± SD. The correlation between the expression of Snail and CHST2 in breast tumor samples was evaluated by the Pearson rank correlation coefficient test. *P* < 0.05 was considered to indicate a statistically significant difference.

## Supplementary information

Supplementary figure legends

Supplementary Figure 1

Supplementary Figure 2

Supplementary Figure 3

Original Data File

aj checklist

## Data Availability

The data, protocols and sequences that support the findings of this study are available from the corresponding authors upon reasonable request.

## References

[1] Friedl P, Wolf K (2003). Tumour-cell invasion and migration: diversity and escape mechanisms. Nat Rev Cancer.

[2] Valastyan S, Weinberg RA (2011). Tumor metastasis: molecular insights and evolving paradigms. Cell..

[3] Zou Y, Ye F, Kong Y, Hu X, Deng X, Xie J (2023). The single-cell landscape of intratumoral heterogeneity and the immunosuppressive microenvironment in liver and brain metastases of breast cancer. Adv Sci.

[4] Mittal V (2018). Epithelial mesenchymal transition in tumor metastasis. Annu Rev Pathol.

[5] Liu P, Wang Z, Ou X, Wu P, Zhang Y, Wu S (2022). The FUS/circEZH2/KLF5/ feedback loop contributes to CXCR4-induced liver metastasis of breast cancer by enhancing epithelial-mesenchymal transition. Mol Cancer.

[6] Wu Y, Zhou BP (2010). Snail: More than EMT. Cell Adhes Migr..

[7] Wang Y, Shi J, Chai K, Ying X, Zhou BP (2013). The role of snail in EMT and tumorigenesis. Curr Cancer Drug Targets.

[8] De Craene B, Berx G (2006). Snail in the frame of malignant tumor recurrence. Breast Cancer Res.

[9] Hojo N, Huisken AL, Wang H, Chirshev E (2018). Snail knockdown reverses stemness and inhibits tumour growth in ovarian cancer. Sci Rep.

[10] Cai F, Xiao H, Sun Y, Wang D, Tang J (2019). Expression of snail and E-cadherin in drug-resistant MCF-7/ADM breast cancer cell strains. J Coll Physicians Surg Pak.

[11] Alba-Castellón L, Olivera-Salguero R, Mestre-Farrera A, Peña R, Herrera M, Bonilla F (2016). Snail1-dependent activation of cancer-associated fibroblast controls epithelial tumor cell invasion and metastasis. Cancer Res.

[12] Li CF, Chen JY, Ho YH, Hsu WH, Wu LC, Lan HY (2019). Snail-induced claudin-11 prompts collective migration for tumour progression. Nat Cell Biol.

[13] Edwards G, Campbell T, Henderson V, Danaher A, Wu D, Srinivasan R (2021). SNAIL transctiption factor in prostate cancer cells promotes neurite outgrowth. Biochimie..

[14] Jordà M, Olmeda D, Vinyals A, Valero E, Cubillo E, Llorens A (2005). Upregulation of MMP-9 in MDCK epithelial cell line in response to expression of the Snail transcription factor. J Cell Sci.

[15] Hsu DS, Lan HY, Huang CH, Tai SK, Chang SY, Tsai TL (2010). Regulation of excision repair cross-complementation group 1 by Snail contributes to cisplatin resistance in head and neck cancer. Clin Cancer Res.

[16] Tao G, Levay AK, Gridley T, Lincoln J (2011). Mmp15 is a direct target of Snai1 during endothelial to mesenchymal transformation and endocardial cushion development. Dev Biol.

[17] Zhang Y, Zou X, Qian W, Weng X, Zhang L, Zhang L (2019). Enhanced PAPSS2/VCAN sulfation axis is essential for Snail-mediated breast cancer cell migration and metastasis. Cell Death Differ.

[18] Stanisavljevic J, Porta-de-la-Riva M, Batlle R, de Herreros AG, Baulida J (2011). The p65 subunit of NF-κB and PARP1 assist Snail1 in activating fibronectin transcription. J Cell Sci.

[19] Uchimura K, El-Fasakhany FM, Hori M, Hemmerich S, Blink SE, Kansas GS (2002). Specificities of N-acetylglucosamine-6-O-sulfotransferases in relation to L-selectin ligand synthesis and tumor-associated enzyme expression. J Biol Chem.

[20] Fujiwara M, Kobayashi M, Hoshino H, Uchimura K, Nakada T, Masumoto J (2012). Expression of long-form N-acetylglucosamine-6-O-sulfotransferase 1 in human high endothelial venules. J Histochem Cytochem.

[21] Hayashida Y, Akama TO, Beecher N, Lewis P, Young RD, Meek KM (2006). Matrix morphogenesis in cornea is mediated by the modification of keratan sulfate by GlcNAc 6-O-sulfotransferase. Proc Natl Acad Sci USA.

[22] Li X, Tu L, Murphy PG, Kadono T, Steeber DA, Tedder TF (2001). CHST1 and CHST2 sulfotransferase expression by vascular endothelial cells regulates shear-resistant leukocyte rolling via L-selectin. J Leukoc Biol.

[23] Grunwell JR, Bertozzi CR (2002). Carbohydrate sulfotransferases of the GalNAc/Gal/GlcNAc6ST family. Biochemistry..

[24] Uchimura K, Kadomatsu K, El-Fasakhany FM, Singer MS, Izawa M, Kannagi R (2004). N-acetylglucosamine 6-O-sulfotransferase-1 regulates expression of L-selectin ligands and lymphocyte homing. J Biol Chem.

[25] Chen L, Ichihara-Tanaka K, Muramatsu T (2004). Role of the carboxyl-terminal region in the activity of N-acetylglucosamine 6-o-sulfotransferase-1. J Biochem.

[26] Uchimura K, Gauguet J-M, Singer MS, Tsay D, Kannagi R, Muramatsu T (2005). A major class of L-selectin ligands is eliminated in mice deficient in two sulfotransferases expressed in high endothelial venules. Nat Immunol.

[27] Kawashima H, Petryniak B, Hiraoka N, Mitoma J, Huckaby V, Nakayama J (2005). N-acetylglucosamine-6-O-sulfotransferases 1 and 2 cooperatively control lymphocyte homing through L-selectin ligand biosynthesis in high endothelial venules. Nat Immunol.

[28] Kawashima H, Fukuda M (2012). Sulfated glycans control lymphocyte homing. Ann N Y Acad Sci.

[29] Girard JP, Amalric F (1998). Biosynthesis of sulfated L-selectin ligands in human high endothelial venules (HEV). Adv Exp Med Biol.

[30] Desko MM, Gross DA, Kohler JJ (2009). Effects of N-glycosylation on the activity and localization of GlcNAc-6-sulfotransferase 1. Glycobiology..

[31] Nandini CD, Sugahara K (2006). Role of the sulfation pattern of chondroitin sulfate in its biological activities and in the binding of growth factors. Adv Pharmacol.

[32] Peinado H, Ballestar E, Esteller M, Cano A (2004). Snail mediates E-cadherin repression by the recruitment of the Sin3A/histone deacetylase 1 (HDAC1)/HDAC2 complex. Mol Cell Biol.

[33] Herranz N, Pasini D, Díaz VM, Francí C, Gutierrez A, Dave N (2008). Polycomb complex 2 is required for E-cadherin repression by the Snail1 transcription factor. Mol Cell Biol.

[34] Hou Z, Peng H, Ayyanathan K, Yan KP, Langer EM, Longmore GD (2008). The LIM protein AJUBA recruits protein arginine methyltransferase 5 to mediate SNAIL-dependent transcriptional repression. Mol Cell Biol.

[35] Lin T, Ponn A, Hu X, Law BK, Lu J (2010). Requirement of the histone demethylase LSD1 in Snai1-mediated transcriptional repression during epithelial-mesenchymal transition. Oncogene..

[36] Okayama H, Kumamoto K, Saitou K, Hayase S, Kofunato Y, Sato Y (2011). Ectopic expression of MECA-79 as a novel prognostic indicator in gastric cancer. Cancer Sci.

[37] Hoshino H, Foyez T, Ohtake-Niimi S, Takeda-Uchimura Y, Michikawa M, Kadomatsu K (2014). KSGal6ST is essential for the 6-sulfation of galactose within keratan sulfate in early postnatal brain. J Histochem Cytochem..

[38] Zhang H, Muramatsu T, Murase A, Yuasa S, Uchimura K, Kadomatsu K (2006). N-Acetylglucosamine 6-O-sulfotransferase-1 is required for brain keratan sulfate biosynthesis and glial scar formation after brain injury. Glycobiology..

[39] Uchimura K, Muramatsu H, Kaname T, Ogawa H, Yamakawa T, Fan QW (1998). Human N-acetylglucosamine-6-O-sulfotransferase involved in the biosynthesis of 6-sulfo sialyl Lewis X: molecular cloning, chromosomal mapping, and expression in various organs and tumor cells. J Biochem.

[40] Henderson V, Smith B, Burton LJ, Randle D, Morris M, Odero-Marah VA (2015). Snail promotes cell migration through PI3K/AKT-dependent Rac1 activation as well as PI3K/AKT-independent pathways during prostate cancer progression. Cell Adhes Migr.

[41] Rosen SD (2004). Ligands for L-selectin: homing, inflammation, and beyond. Annu Rev Immunol.

[42] Hoshino H, Ohta M, Ito M, Uchimura K, Sakai Y, Uehara T (2016). Apical membrane expression of distinct sulfated glycans represents a novel marker of cholangiolocellular carcinoma. Lab Investig.

[43] Taga M, Hoshino H, Low S, Imamura Y, Ito H, Yokoyama O (2015). A potential role for 6-sulfo sialyl Lewis X in metastasis of bladder urothelial carcinoma. Urol Oncol.

[44] Guerrero PE, Miró L, Wong BS, Massaguer A, Martínez-Bosch N, Llorens R (2020). Knockdown of α2,3-sialyltransferases impairs pancreatic cancer cell migration, invasion and E-selectin-dependent adhesion. Int J Mol Sci.

[45] Dall’Olio F, Pucci M, Malagolini N (2021). The cancer-associated antigens sialyl Lewis(a/x) and Sd(a): two opposite faces of terminal glycosylation. Cancers.

[46] Bowman KG, Cook BN, de Graffenried CL, Bertozzi CR (2001). Biosynthesis of L-selectin ligands: sulfation of sialyl Lewis x-related oligosaccharides by a family of GlcNAc-6-sulfotransferases. Biochemistry..

[47] Pinho SS, Reis CA (2015). Glycosylation in cancer: mechanisms and clinical implications. Nat Rev Cancer.

[48] Mitoma J, Bao X, Petryanik B, Schaerli P, Gauguet JM, Yu SY (2007). Critical functions of N-glycans in L-selectin-mediated lymphocyte homing and recruitment. Nat Immunol.

[49] Yeh JC, Hiraoka N, Petryniak B, Nakayama J, Ellies LG, Rabuka D (2001). Novel sulfated lymphocyte homing receptors and their control by a Core1 extension beta 1,3-N-acetylglucosaminyltransferase. Cell..

[50] Li Q, Peng H, Fan H, Zou X, Liu Q, Zhang Y (2016). The LIM protein Ajuba promotes adipogenesis by enhancing PPARγ and p300/CBP interaction. Cell Death Differ.

[51] Qian W, Li Q, Wu X, Li W, Li Q, Zhang J (2020). Deubiquitinase USP29 promotes gastric cancer cell migration by cooperating with phosphatase SCP1 to stabilize Snail protein. Oncogene.

[52] Rodriguez LG, Wu X, Guan JL (2005). Wound-healing assay. Methods Mol Biol.

