# Supplementary material for: CHST2-mediated sulfation of MECA79 antigens is critical for breast cancer cell migration and metastasis
Source: Cell Death Dis. 2023 Apr 24;14(4):288. doi: 10.1038/s41419-023-05797-x (PMC10126008; doi:10.1038/s41419-023-05797-x)
Supplement: Supplementary file 1 — Supplementary figure legends [file 41419_2023_5797_MOESM1_ESM.docx]

**Supplement Figures**

**Supplemental 1 Snail activates CHST2 transcription through binding DNA elements resided in CHST2 proximal promoter.**

A The diagram shows two putative binding sites of Snail in CHST2 promoter were mutated. B Wild-type or mutant CHST2 promoter-driven luciferase reporter plasmids were co-transfected with pGL3-Snail plasmids into 293T cells. Transfected cells were harvested for luciferase reporter assays and the value was normalized to β−gal (Three repeats), *P < 0.05, ns, no significance. Students’ t test. C The ChIP assays were performed in MCF-0A-Snail cells with speciﬁc antibody against Snail and the enriched DNA fragments were examined by qPCR. Error bars show standard deviations. **P<0.01, ns means no significance.

**Supplemental 2 The sulfur supply is required for CHST2 to induce cell migration.**

A Immunoblots showed CHST2 expression levels in MCF-7-CHST2 cells. B Transwell assays showed migration capabilities of CHST2-overexpressed MCF-7 cells in the presence of 0, 5, 10 mM sodium chlorate for 48 h. six fields chosen randomly were counted for statistical analysis. Data were shown as mean ± S.D. from three independent experiments, *P < 0.05, **P < 0.01, ns, no significance. Students’ t test. C Cell viabilities of MCF-10A-CHST2 cell were shown in the treatment of different concentration of sodium chlorate for 48 h, n = 6 biologically independent samples, unpaired two-tailed t test. D Immunoblots showed CHST2 protein levels in MCF-10A-CHST2 cells after treatment with sodium chlorate for 48 h.

**Supplemental 3 CHST2 is an essential target gene for Snail-induced cell migration**.

A Transwell assays showed migration capabilities of Snail-overexpressed MCF-7 cells in the presence of 0, 5, 10 mM sodium chlorate for 48 h. Six fields chosen randomly were counted for statistical analysis. Data were shown as mean ± S.D. from three independent experiments (right panel), *P < 0.05, **P < 0.01. Student t test. B Cell viability (%) of MCF-7 cells stably expressing vector or Snail treated with vehicle, sodium chlorate (0, 5, 10 mM) for 48 h. For cell viability assays, data are mean ± S.D., n = 5 biologically independent samples, unpaired two-tailed t test.
