# Supplementary figures and images for: CHST2-mediated sulfation of MECA79 antigens is critical for breast cancer cell migration and metastasis
Source: Cell Death Dis. 2023 Apr 24;14(4):288. doi: 10.1038/s41419-023-05797-x (PMC10126008; doi:10.1038/s41419-023-05797-x)

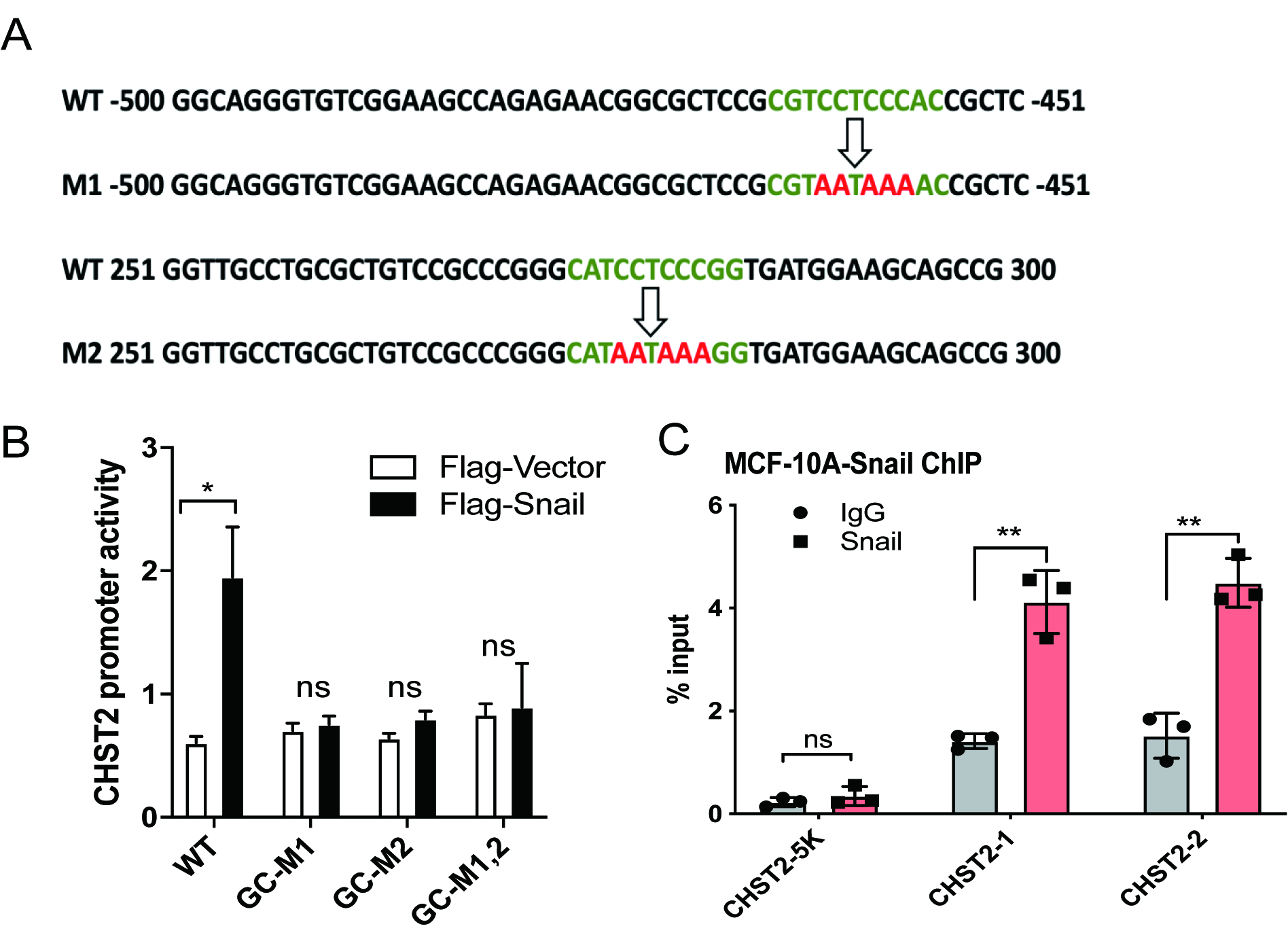

Supplement: Supplementary file 2 — Supplementary Figure 1 [file 41419_2023_5797_MOESM2_ESM.tif]

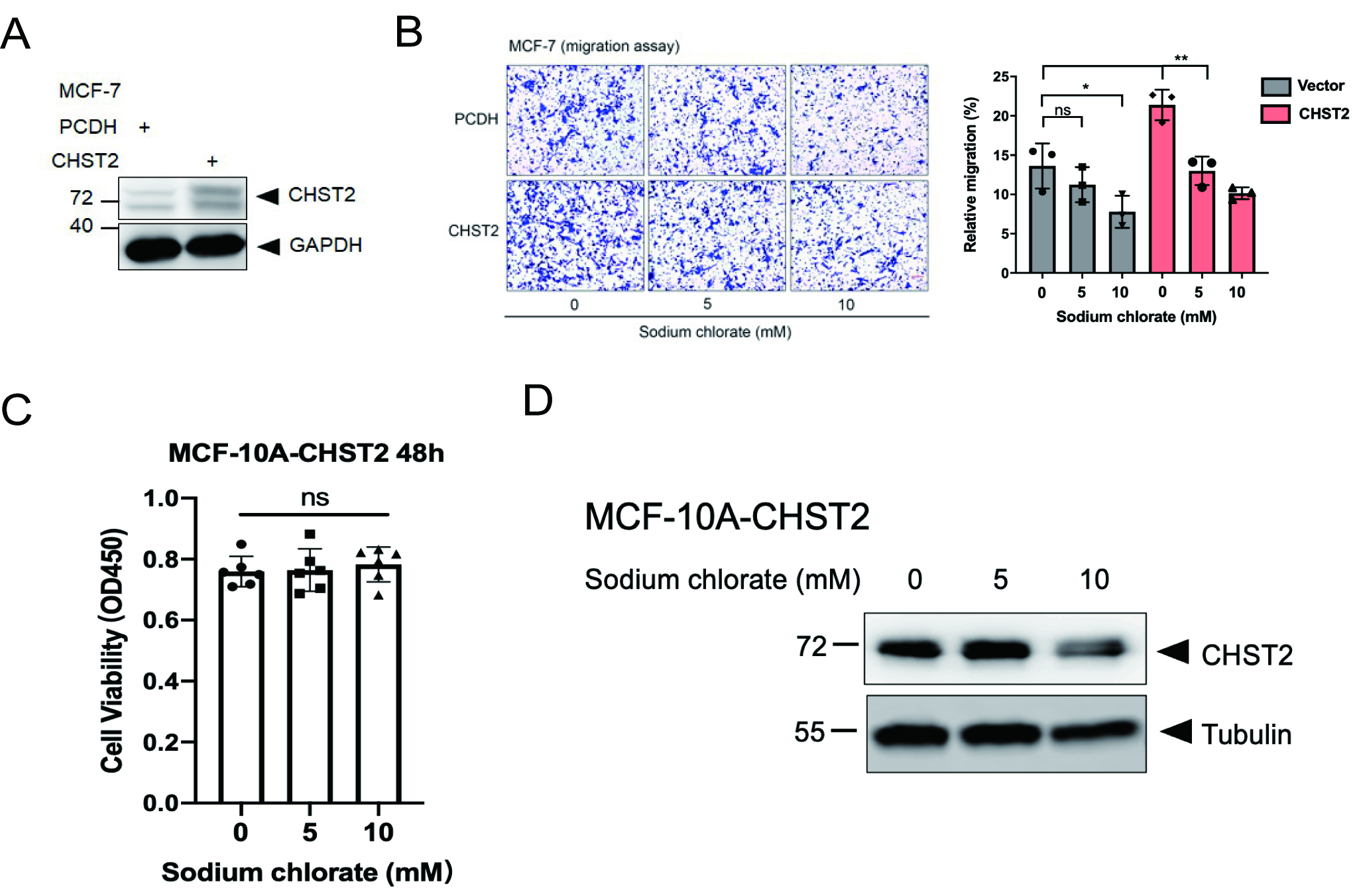

Supplement: Supplementary file 3 — Supplementary Figure 2 [file 41419_2023_5797_MOESM3_ESM.tif]

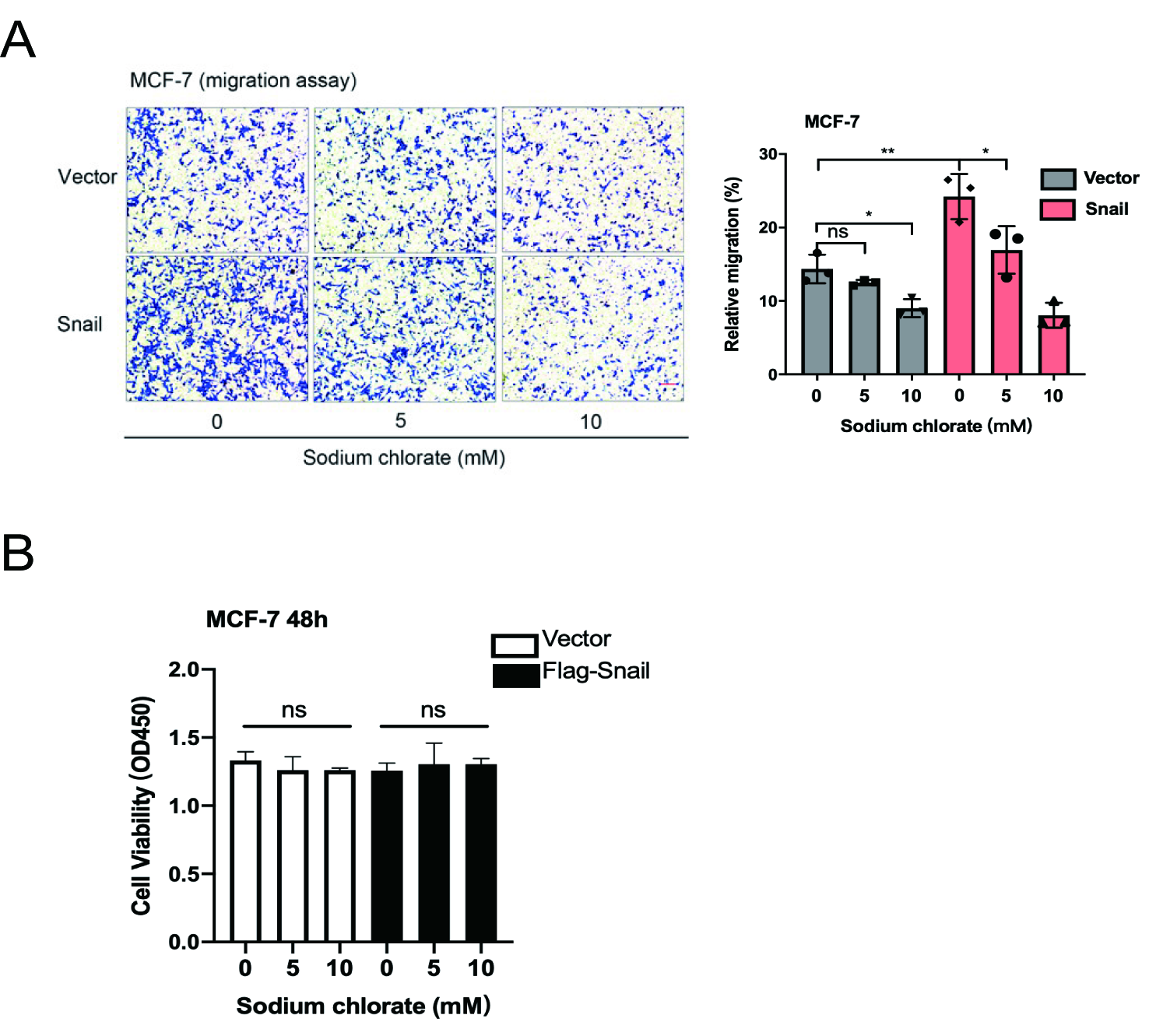

Supplement: Supplementary file 4 — Supplementary Figure 3 [file 41419_2023_5797_MOESM4_ESM.tif]
