# Supplementary material for: CHST2-mediated sulfation of MECA79 antigens is critical for breast cancer cell migration and metastasis
Source: Cell Death Dis. 2023 Apr 24;14(4):288. doi: 10.1038/s41419-023-05797-x (PMC10126008; doi:10.1038/s41419-023-05797-x)
Supplement: Supplementary file 5 — Original Data File [file 41419_2023_5797_MOESM5_ESM.docx]

Original Western blots：

Figure 1C：
Figure 1D:

 Figure 1E:

Figure 1G:

Figure 2A：


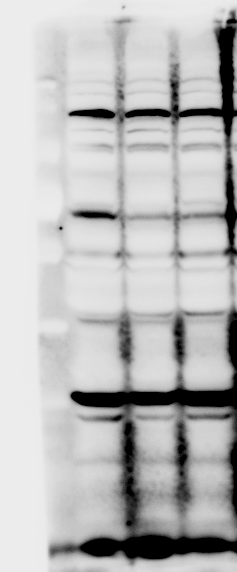

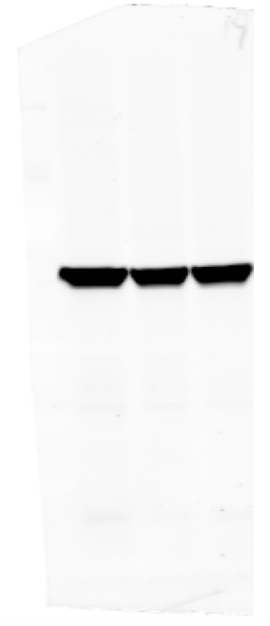


CHST2

Tubulin

Figure 2D：


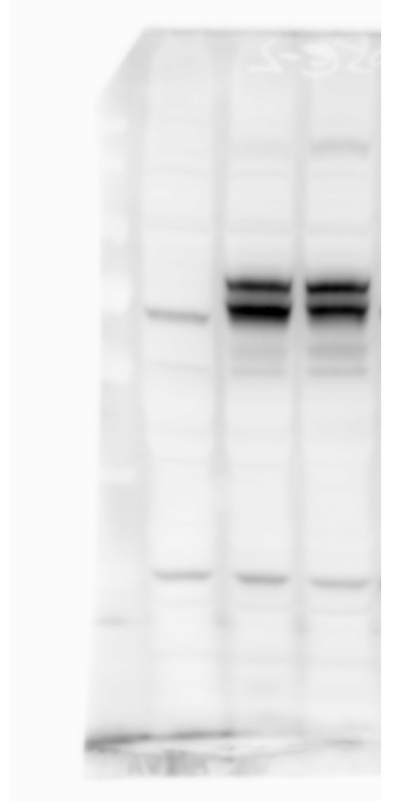


CHST2


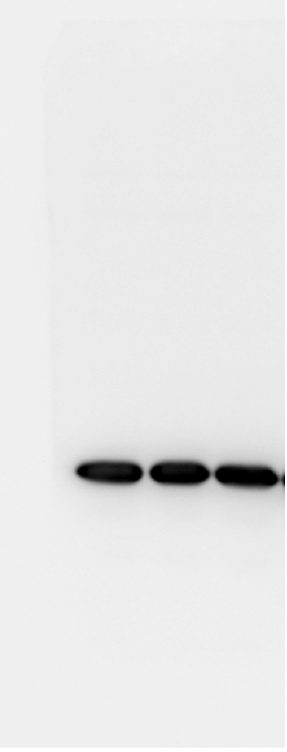


GAPDH

Figure 3C：


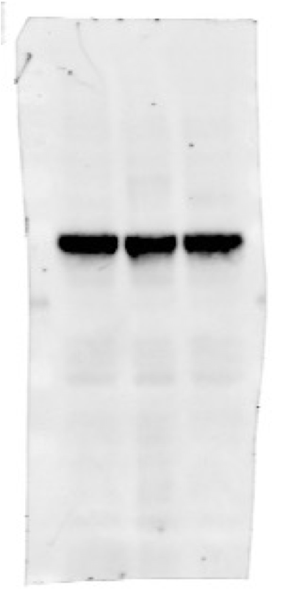


Tubulin


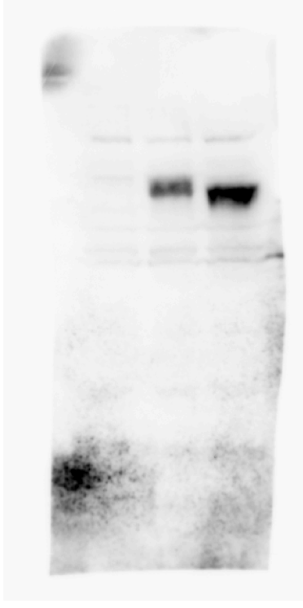


CHST2

Figure 5A：


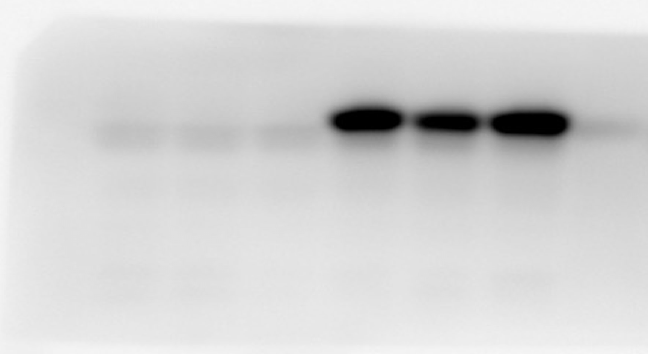


Snail


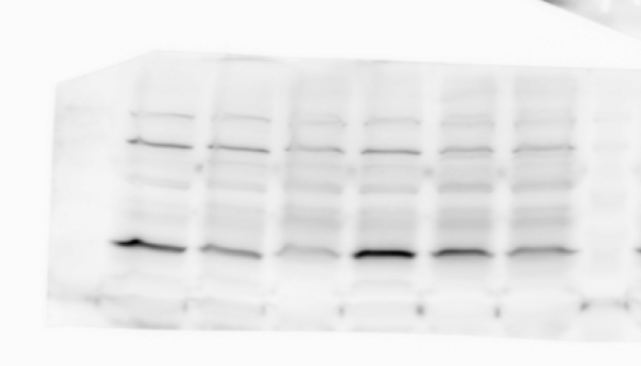


CHST2


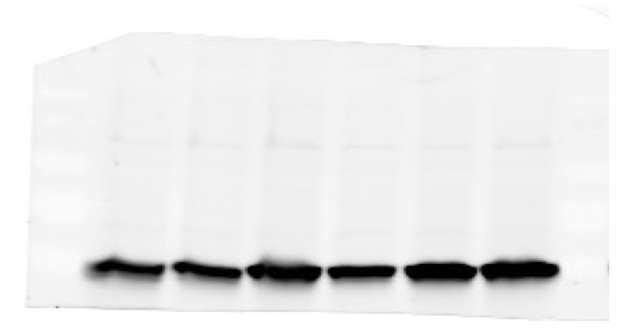


Tubulin

Figure 5C：


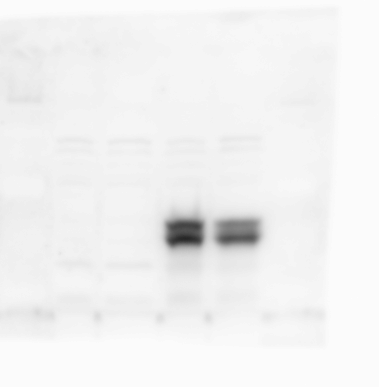


CHST2


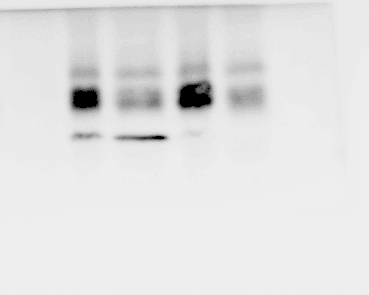


Snail


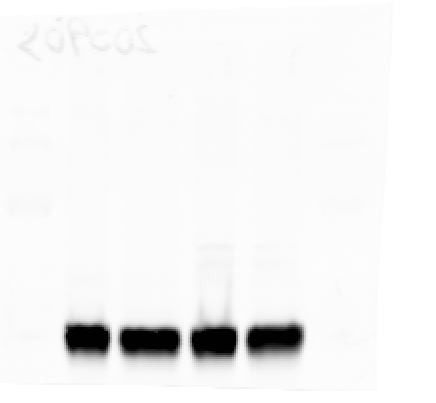


Tubulin

Supplemental figure 1A


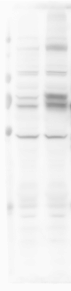

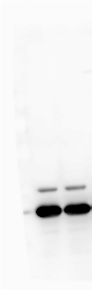


GAPDH

CHST2

Supplemental figure 1D


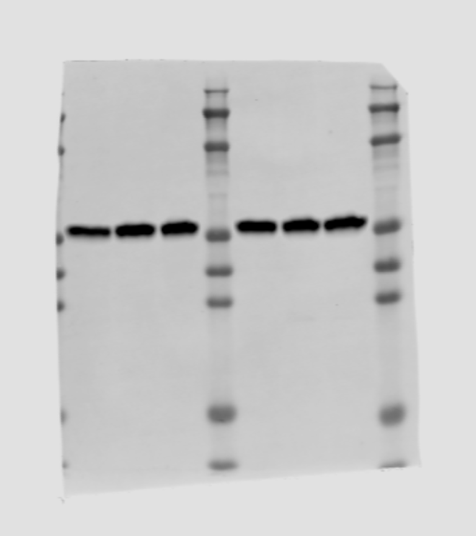


Tubulin


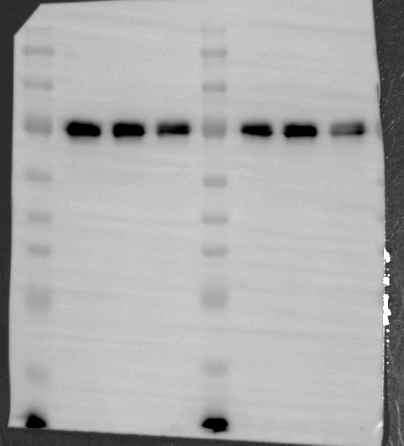


CHST2
